# The Importance of Incorporating Landscape Change for Predictions of Climate-Induced Plant Phenological Shifts

**DOI:** 10.3389/fpls.2020.00759

**Published:** 2020-06-25

**Authors:** Chelsea Chisholm, Michael S. Becker, Wayne H. Pollard

**Affiliations:** ^1^Center for Macroecology, Evolution and Climate, Natural History Museum of Denmark, University of Copenhagen, Copenhagen, Denmark; ^2^Department of Geography, McGill University, Montreal, QC, Canada

**Keywords:** leaf phenology, flower phenology, permafrost, geomorphology, ground stability, Arctic, plant ecology

## Abstract

Warming in the high Arctic is occurring at the fastest rate on the planet, raising concerns over how this global change driver will influence plant community composition, the timing of vegetation phenological events, and the wildlife that rely on them. In this region, as much as 50% of near-surface permafrost is composed of thermally sensitive ground ice that when melted produces substantial changes in topography and microbiome conditions. We take advantage of natural variations in permafrost melt to conduct a space-for-time study on Ellesmere Island in northern Canada. We demonstrate that phenological timing can be delayed in thermokarst areas when compared to stable ground, and that this change is a function of shifting species composition in these vegetation communities as well as delayed timing within species. These findings suggest that a warming climate could result in an overall broadening of blooming and leafing windows at the landscape level when these delayed timings are taken into consideration with the projected advance of phenological timings in ice-poor areas. We emphasize that the impacts of geomorphic processes on key phenological drivers are essential for enhancing our understanding of community response to climate warming in the high Arctic, with implications for ecosystem functioning and trophic interactions.

## Introduction

The high Arctic is warming at twice the global average and is anticipated to have significant effects on the landscape, flora, and fauna of the region (IPCC, 2014). Warming air temperatures have already resulted in the increased thawing of permafrost and accelerated melting of ground ice (Callaghan et al., 2011; Lewkowicz and Way, 2019). It is predicted that with increased ground and air temperatures there will be large shifts in the region’s flora (Elmendorf et al., 2012a) with consequences for both ecosystem functioning (Schuur and Mack, 2018) and the carbon balance (Turetsky et al., 2020). One of the key floral changes predicted is altered phenological timings, particularly advancing flowering and peak green-up dates (Arft et al., 1999; Høye et al., 2007; Oberbauer et al., 2013; Prevéy et al., 2019). The sensitivity of plant communities to phenological change is also expected to be greater in cold, high latitude sites than warmer sites (Prevéy et al., 2017). Variation in phenology can have strong influences on competitive interactions and species coexistence (Wolkovich and Cleland, 2011) and there are important concerns regarding how these altered life-cycle timings may result in trophic mismatch with insect pollinators (Høye et al., 2014; Schmidt et al., 2016) and migratory herbivores (Doiron et al., 2015).

There have only been a few long-term studies of climate-induced phenological effects in a natural high Arctic setting (e.g., Bjorkman et al., 2015; Høye and Forchhammer, 2008) due to the area’s remoteness and the investment required for field collection in Arctic environments (Martin et al., 2012; Metcalfe et al., 2018). In order to overcome these challenges, studies have often utilized passive warming devices such as *in situ* experimental warming chambers to simulate the effects of climate change on plant communities (Henry and Molau, 1997; Elmendorf et al., 2012b) and plant traits (Baruah et al., 2017; Bjorkman et al., 2018). These open-top chamber (OTC) studies have demonstrated a range of effects on plants, including changes in aboveground productivity, altered species dominance, and shifting biodiversity, with strong regional variation observed in the direction of response (Elmendorf et al., 2012a). In particular, experiments utilizing OTCs have demonstrated that high Arctic phenology is especially sensitive to warming temperatures (Prevéy et al., 2017), though interestingly these responses often differ at the species- or functional group-level (Arft et al., 1999; Cooper, 2014; Prevéy et al., 2019). Previous research has found support for the correspondence between observational data and OTC studies for some plant community response variables (e.g., abundance changes; Elmendorf et al., 2015). However, this has not been the case for phenological response to warming. Wolkovich et al. (2012) demonstrated that experimental studies consistently underpredicted the response of phenology to warming, as compared to long-term studies. They suggest this may be due to artifacts introduced by the chambers themselves, as well as other proximate drivers of phenology change that are not captured through experimental warming studies.

One such neglected driver relates to the response of near-surface ground ice to climate change. Permafrost landscapes display a high degree of topographic irregularity associated with buried ground ice and the dynamics of seasonally thawed ground, generally called the active layer. The active layer is important as seasonal freeze-thaw cycles result in patterned ground features that can be highly reactive to variations in air temperature, with impacts ranging from small downward shifts in relief of a few centimeters, to ground slides and slumps measured in meters (Pollard, 2017). Up to 50% of the volume of the top 3m of ground in the high Arctic may be composed of ground ice (Pollard and French, 1980; Couture and Pollard, 1998, 2007; Liljedahl et al., 2016) mainly in the form of ice wedges. Ice wedges are a v-shaped body of ice and are a ubiquitous feature of permafrost environments, found within up to 25% of the Earth’s terrestrial surface (Zhang et al., 1999). An increase in seasonal thaw can result in ice wedge melt (thermokarst), which depresses the overlying trough soil (subsidence; Liljedahl et al., 2016) and creates a highly patterned landscape of interconnected polygons with shallow troughs underlain by ice wedges (Pollard, 2017). The subsidence of ice wedges increases winter snowpack depth and the collection of surface water in an otherwise water-limited environment. This new moisture regime promotes plant growth that insulates the ground from warm summer temperatures, resulting in shallower active layers and overall colder ground temperatures throughout the growing season (Shur and Jorgenson, 2007).

A major reason for the mismatch between phenological observations and experimental warming results in the high Arctic may be the neglect of key geomorphic changes predicted to occur concurrently with climate warming. Climate change has increased rates of thermokarst in this region (Ward Jones et al., 2019) and this is predicted to continue unabated into the future (Jorgenson et al., 2015). Thermokarst is expected to drive down soil temperatures through complex hydrological interactions, resulting in the recruitment of wetland vegetation to replace traditionally polar desert habitats (Becker et al., 2016). Phenology studies employing passive warming methods generally sample from stable ground surfaces, thereby ignoring relevant factors such as changes to surface hydrology (Woo and Young, 2006), active layer depth (seasonal depth of thaw) (Jiang et al., 2012), and ground stability (Jorgenson et al., 2015). The question remains as to how geomorphological drivers may affect leafing and flowering times, and whether these results agree with previous syntheses of phenology studies in regions commonly underlain by permafrost. Given that thawed permafrost and melting ground ice result in a substantial divergence in vegetation community composition (Jorgenson et al., 2015; Becker et al., 2016), another major question is whether changes in phenology at the landscape scale are driven by changes in within-species timing or by plant community turnover resulting from the creation of thermokarst wetlands.

This study examines how geomorphologic processes act to drive phenological response in high Arctic plant communities. We adopted a space-for-time approach using both species- and plot-level measures of phenology across a thermokarst gradient to examine the influence of geomorphological change on phenology. We conducted this research at a site of naturally occurring climate-induced thermokarst on the Fosheim Peninsula, Ellesmere Island, Nunavut within the Canadian high Arctic (Pollard, 2000). This region has experienced recent widespread initialization of thermokarst activity over the past decade due to climate warming (Ward Jones et al., 2019), stressing the importance of understanding how thermokarst will impact both plant communities and their life cycles. We compared changes in a suite of phenological traits across plant communities in both undisturbed polar desert and thermokarst terrain, as well as differences across polygon features created by ice wedge degradation (elevated tops vs subsided troughs). More specifically, we examined how the development of thermokarst drives changes in community-level phenology as well as the intraspecific variability in phenology of a widespread species, *Salix arctica*. We predicted the following: (1) thermokarst troughs would experience colder ground temperatures due to the subsidence of ice wedges and associated abiotic changes (as seen in Becker et al., 2016) and (2) the phenology of vegetation in thermokarst areas would be delayed in comparison to non-thermokarst terrain due to thermokarst-driven temperature shifts (Arft et al., 1999). As thermokarst is responsible for strongly divergent microhabitat conditions that not only directly affect phenology, but also result in large biodiversity shifts across these steep gradients (Zona et al., 2011), we further examined the contribution of species turnover and intraspecific variability to phenological responses.

## Materials and Methods

**FIGURE 1:**
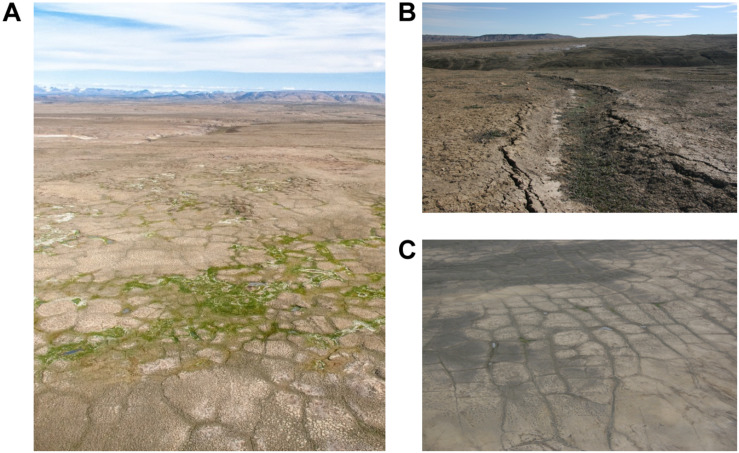
**(A)** Photo depicting an area of thermokarst (ground ice melt) on the Fosheim Peninsula, Ellesmere Island, Nunavut, Canada. The photograph shows the study site during the summer season, where thermokarst and adjacent polar desert polygons were sampled. **(B)** Photo depicting a polygon trough. **(C)** This photo highlights the presence of polygon features across the landscape, showing ice wedge polygons with high relief tops and low relief troughs.

This study was conducted at a site of climate-induced thermokarst in the high Arctic on the Fosheim Peninsula of Ellesmere Island, Nunavut, Canada (79.84574°N, 85.37028°W). This region is characteristic of a polar desert environment with little precipitation, nutrient poor soils, and an extremely short growing season. The Fosheim experiences an exceedingly cold mean annual air temperature (MAAT) of −18.8°C with summer temperatures only reaching an average of 6.1°C in July (Environment Canada, 2010). These cold conditions result in deep permafrost that is over 500 m thick (Taylor, 1991), exceptionally thin average seasonal ground thaw compared to other areas of the Arctic (mean active layer of 57 cm; Couture and Pollard, 2007), and a small species pool estimated at ∼140 vascular plant species (Edlund et al., 1990). There is ∼1456.8 km^2^ of ground ice in the Fosheim Peninsula alone, of which ∼700 km^2^ likely consists of wedge ice (Couture and Pollard, 1998) though recent satellite based measurements have estimated that ice wedges could occur across 50% of the land cover of the peninsula, or ∼3000 km^2^ (Bernard-Grand’Maison and Pollard, 2018). We selected a 200 m × 100 m study site (panel A of Figure 1) to be characteristic of the general landscape of the Fosheim Peninsula, with geomorphic and vegetation differences at the site representative of localized thermokarst processes predicted to increase with a warming climate as outlined in Becker et al. (2016). Panels B and C of Figure 1 further demonstrate the differences in microtopography encountered in this region, with panel B illustrating subsidence due to ice wedge degradation (producing a polygon trough), and panel C showing the typical polygonal patterning found in these ground ice-dominated landscapes.

### Data Collection

As thermokarst causes an abrupt disturbance in temperature regime and ground stability (Jorgenson et al., 2015), we used microtopography as a natural experiment in which to assess whether differences in abundance-weighted phenology were due more to intraspecific variability or changes in the abundance and identity of species in the community. Phenological data and ground temperature data for this study were collected in concert with abiotic and biotic data collected for a separate study during the polar summer of 2013 (see Becker et al. (2016) for further methodological details). We chose a study site containing a thermokarst wetland in order to sample an area undergoing climate-induced permafrost melt and the adjacent polar desert terrain. Both of these ground states exhibit polygonal microtopographies, with higher relief “tops” and lower relief “troughs.” We established plots immediately after snowmelt using a stratified sampling approach along five transects running 200 m east to west, spaced 25 m apart north to south. We systematically sampled each polygon top and trough feature traversed by the transects, placing 0.5 m^2^ plots every 5–10 m at alternating polygon tops and troughs (allowing for some spatial variation due to differences in the sizes of each polygon feature traversed). Our design is fully factorial, with plots located in either polar desert or thermokarst ground states, and top or trough polygon features. We hereafter refer to the polar desert ground state as the control state, as we are interested in the shift in phenology due to an increase in thermokarst in this region. This resulted in 80 plots in total, with 20 plots each across each of four habitat categories: control (polar desert) top, control (polar desert) trough, thermokarst top and thermokarst trough. However, we report 75 plots in our results as there were five plots found in thermokarst troughs which remained submerged with water throughout the growing season. We visually estimated community composition and relative percent cover within each plot at approximately peak biomass. All vascular vegetation was identified to the species level, with nomenclature following Saarela et al. (2013) (see Supplementary Table S1 for species information).

We recorded hourly temperature changes through the summer season and computed total thaw degree days (TDD) using Onset HOBO data loggers buried at a depth of 10 cm (approximating maximum rooting depth) in each plot. TDD is a measure of the magnitude of warming above 0°C. To calculate TDD, we averaged temperature values to gain a daily mean temperature for each plot and summed mean temperatures for all days greater than 0°C. We chose to use TDD in our analyses, and not growing degree days (GDD, or cumulative temperature >5°C, the temperature at which plants generally experience growth), as our focal species are selected for growing in cold conditions and some of the phenophase dates observed occurred before plots exhibited GDD values greater than 0. Data loggers were installed on June 28th immediately after snowmelt and retrieved on July 20th. Some ground thaw had already occurred in order to allow for sufficient burial depth (the ground freezes to the surface every winter); as such we consider our TDD metric as the relative magnitude in temperature experienced between microtopographies for the summer, rather than the absolute TDD for the summer period.

Phenological trait measures of both flowering and leaf emergence and growth were assessed within each plot for all vascular plant species. We recorded the following phenological traits as measures: (first) initial leaf growth, (first) full leaf out, (first) flower bud (including flower heads for grasses/sedges) and (first) open flowering, using methodology detailed in the United States National Phenology Network (NPN) (Denny et al., 2014). These phenophases capture a broad range of phenological responses that may be affected by thermokarst and have different implications for interactions between trophic levels (i.e., delayed flowering time may affect pollinators more, whereas delayed leafing may preferentially impact herbivores). Phenological measures were sampled every second day in each plot during the field season. Because many species within our plots are clonal and it is difficult to differentiate individuals, we recorded plot-level phenophase measures for each species.

### Statistical Analysis of Phenological Data

All statistical analyses and graphics were conducted in R version 3.6.3 (R Core Team, 2020), using the package tidyverse for data wrangling (Wickham et al., 2019) and the packages ggthemes (Arnold, 2019) and cowplot (Wilke, 2019) for graphics.

In order to compare changes in community phenological responses, we calculated weighted average Julian dates for flower bud, open flowers, initial leaf growth, and full leaf out across all species within each plot. Weighted averages were based on community relative abundances generated using the function “decostand” in the R package vegan (Oksanen et al., 2019). We compared abundance weighted phenological timing using two-way ANOVAs with type II sum of squares using the car package (Fox and Weisberg, 2019), as sample number differed based on microtopography, and compared effects of top vs trough and control vs thermokarst using the function “emmeans” in the R package emmeans (Lenth, 2020), with all pairwise comparisons corrected using a tukey *p*-value adjustment. We additionally used the package nlme (Pinheiro et al., 2020) to test for the effect of spatial variation on our results. We performed the same analyses as above using linear mixed effects models with “transect” as a random effect and assessed whether there was statistical support for the inclusion of this as a random effect in our models. As we were also interested in evaluating within-species changes in phenology, we chose one species, *S. arctica*, to compare phenology traits across microtopography using a type II ANOVA. This species was selected as it was found in 80% of censused plots at this study site (it being the only species occupying >50% of plots censused) and was the most abundant species across the site, with an average of 60% cover in occupied plots. We performed the same statistical tests as described above, assessing differences in phenophase traits of *S. arctica* (non-weighted) as predicted by ground state, polygon feature, and their interaction. We assessed importance of these predictors using both type II ANOVAs and linear mixed effects models, as described previously.

To tease apart the predominant contributors to community-averaged phenological timings, we decomposed variation in flowering and leafing dates into interspecific (species compositional turnover) and intraspecific (within-species variation) components across microtopography, following code adapted from Lepš et al. (2011). This approach is particularly suited to our study, as there are large species compositional differences in plant communities in thermokarst and polar desert habitats. Here we use “fixed” and “specific” averages to describe whether differences in mean trait variability between treatments at the plot-level are due to a change in species composition or in intraspecific trait changes. We first calculated fixed averages in phenological timings per species across the entire site, which is a single mean trait value for that species. Using only these fixed averages, we generated relative abundance weighted averages for each plot. Therefore, any observed differences in fixed averages can only be due to compositional change, or species turnover in plant communities. Specific averages used individual observations for each species within each plot to calculate a relative abundance weighted mean, and are the same averaged phenophase data that were compared in our previous analyses. The difference between fixed averages and specific averages removes the effect of species composition change, or turnover, and as such can be solely attributed to intraspecific variability in phenological timing. The response of phenophase timing to microtopography was analyzed using a two-way type II ANOVA, decomposed into the sum of squares of species turnover, intraspecific variability, and their covariation. See further details on this methodology in the Supplementary Information (Methods S1).

In order to assess the effects of temperature on phenology at our study site, we first tested overall differences in TDD across the summer season using a two-way type II ANOVA to account for unequal sample sizes. To test the effect of TDD on phenophases, we calculated a cumulative TDD for all days prior to the Julian date at which the phenophase trait commenced for all four phenophases tested. As we were testing relative abundance weighted means of phenophases, we rounded down the averaged number and generated a cumulative sum of average daily temperatures >0°C up to that whole Julian date, such that each plot and phenophase trait was attributed a unique TDD value. We analyzed each phenophase using linear models with the plot- and phenophase-specific TDD value as a predictor. As we were interested in whether ground state and polygon feature explained any residual variation after TDD was accounted for, we performed an ANCOVA analysis with TDD as the first covariate and polygon feature, ground state and their interaction as secondary predictors. This analysis controls for the effect of a covariate TDD and tests whether there is any remaining effect of our two treatments which were not explained by temperature.

We transformed data using a square root transformation for the response community-weighted initial leaf growth dates and a ln transformation for the predictor cumulative temperature where appropriate.

## Results

Data loggers were successfully retrieved on July 20th after 22 days in the ground (data inclusive of Julian dates 179–200), capturing the full window between early spring (before leaf growth or flowering commenced) and summer season. Across the study site, this particular summer was characterized by a late snowmelt on June 25th, colder than average July temperature of 4.4°C, and an early autumn snowfall on August 12th. This later snowmelt and early snowfall created a particularly short summer window for plant life to accomplish the typical lifecycle processes of inflorescence bloom, leaf growth, energy capture and storage, reproduction, and senescence. We found strong, significant differences in plot ground temperature for the different microtopographies. Thermokarst plots experienced significantly lower TDD for the study period than control areas (95.3 (SE 3.54) and 119.2 (SE 3.32), respectively; *F*_1,__70_ = 23.7, *P* < 0.001), with polygon troughs having lower TDD than polygon tops (91.5 (SE 3.54) and 122.9 (SE 3.32), respectively; *F*_1,__70_ = 41.2, *P* < 0.001) and no significant interaction effect (*F*_1,70_ = 1.12, *P* = 0.294; see panel A of Supplementary Figure S1).

**FIGURE 2:**
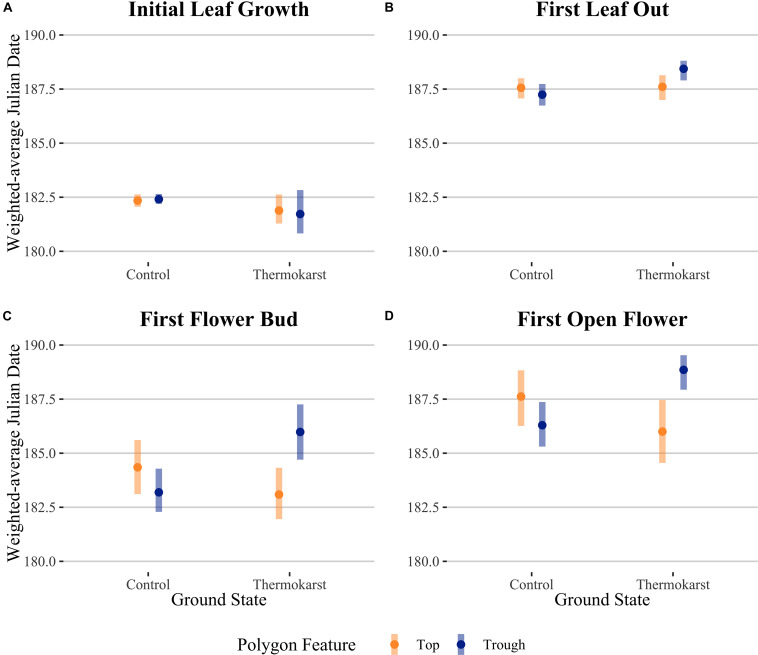
Abundance-weighted average Julian dates for community-level **(A)** initial leaf growth, **(B)** first leaf out, **(C)** first flower bud and **(D)** first open flower across ground state (control vs thermokarst), with colors denoting polygon feature (top = orange vs trough = blue) categories. Points denote mean values, while bars represent 95% confidence intervals.

Rather than being dominated only by *S. arctica* (as in the control plots), thermokarst plots were generally dominated by high-moisture preference species such as *Dupontia fisheri* and *Carex aquatilus*, in addition to *S. arctica* (Supplementary Figure S2). The entire species pool of the site was limited to 22 out of an estimated 140 indigenous to the area (see Supplementary Table S1) (Edlund et al., 1990), with five species unique to control plots and five species unique to thermokarst areas. Weighting phenophase traits by relative abundance, we found a significant interaction effect between polygon feature and ground state for first leaf out, first flower bud, and first open flowers timings (Figure 2; see Table 1 for associated statistics). We found no significant differences between polygon tops in thermokarst or control plots for any of the phenophases. Compared to control troughs, thermokarst troughs had significantly or marginally significant delayed leaf out (1.20 days (SE 0.395), *t_67_* = −3.03, *P* = 0.018), flower bud (2.29 days (SE 0.98), *t_64_* = −2.31, *P* = 0.099), and open flowers (2.56 days (SE 0.94), *t_60_* = −2.74, *P* = 0.039). Thermokarst troughs also exhibited delayed phenology as compared to thermokarst tops, with later flower bud (3.16 days (SE 1.02), *t_64_* = −3.11, *P* = 0.015) and open flowers (2.60 days (SE 0.97), *t_60_* = −2.69, *P* = 0.045). These significant delays (between 1.20 and 3.12 days) in three key phenological traits in plant communities found in depressed thermokarst troughs as opposed to thermokarst tops represent a 6.7–14.9% shift in timing across the growing season. The only trait to differ from these overall trends was initial leaf growth, with no significant effects of ground state, polygon feature, or their interaction.

**TABLE 1:** Abundance-weighted Type II ANOVA results for all four phenological timing response variables.

**Response**	**Effect**	**SS**	***F***	***P***
Initial leaf growth	Ground state	5.9	3.345	0.072
	Feature	0	0.018	0.895
	Interaction	0.2	0.130	0.719
First leaf out	**Ground state**	**6.0**	**4.705**	**0.034**
	Feature	0.6	0.458	0.501
	**Interaction**	**5.7**	**4.465**	**0.038**
First flower bud	Ground state	1.9	0.244	0.623
	Feature	18.6	2.412	0.125
	**Interaction**	**59.5**	**7.720**	**0.007**
First open flower	Ground state	3.7	0.569	0.454
	Feature	2.3	0.349	0.557
	**Interaction**	**60.0**	**9.338**	**0.003**

*Significant *P-*values (<0.05) are in bold.*

**FIGURE 3:**
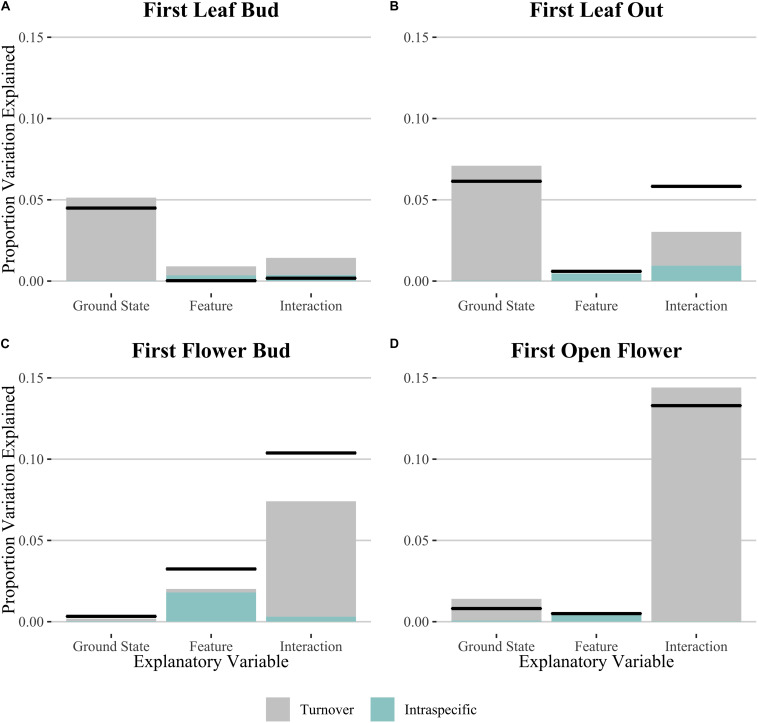
Relative Sum of Squares obtained from variance decomposition analysis of the effect of ground state, polygon feature and their interaction on timing of phenophases: **(A)** initial leaf growth, **(B)** first leaf out, **(C)** first flower bud and **(D)** first open flower. Black lines denote total variation explained for each explanatory variable. Colored bars denote the proportional variance contribution for both species turnover (Turnover; gray) and intraspecific variability (Intraspec.; blue). See Table 2 for associated statistics.

In order to test whether spatial variation may play a role in the results we found, we performed the same analyses using linear mixed effects models including transect as a random effect (Supplementary Table S2). We found no statistical support for including the random effect in our models for those phenophase traits which showed a significant effect of microtopography on Julian date, with the simple linear model showing consistently lower AIC values. We then used the linear models to decompose variation due to intraspecific change and community turnover for all phenophase traits (Figure 3 and Table 2). Species turnover comprised the greatest contribution to variation in phenophase explained by ground state and the interaction between ground state and polygon feature. For first leaf out and first flower bud, there was a large positive covariation between turnover and intraspecific contributions to the interaction of ground state and feature, whereas covariation in the interaction term was smaller and negative for initial leaf growth and open flowers.

**TABLE 2:** Variance decomposition results for **(A)** initial leaf growth, **(B)** first leaf out, **(C)** first flower bud and **(D)** first open flower phenology measures.

	**Turnover**	**Intraspecific**	**Covariation**	**Total**
**(A) Initial leaf growth**				
Ground state	**6.7**	0	−0.8	5.9
Feature	0.7	0.5	−1.2	0
Interaction	1.4	0.5	−1.7	0.2
Residuals	66.8	50.2	8.2	125.2
Total	75.6	51.2	4.5	131.3
**(B) First leaf out**				
Ground state	**7.0**	0	−1	**6.0**
Feature	0	0.5	0.1	0.6
Interaction	**2.0**	0.9	2.8	**5.7**
Residuals	20.6	70.8	−5.3	86.1
Total	29.6	72.2	−3.4	98.4
**(C) First flower bud**				
Ground state	0.9	0.2	0.8	1.9
Feature	1.2	**10.3**	7.1	18.6
Interaction	**40.7**	1.8	17.0	**59.5**
Residuals	387.0	114.0	−7.7	493.3
Total	429.8	126.3	17.2	573.3
**(D) First open flower**				
Ground state	6.0	0.3	−2.6	3.7
Feature	0	2.3	0	2.3
Interaction	**65.0**	0.1	−5.1	**60.0**
Residuals	333.3	54.7	−2.2	385.8
Total	404.3	57.4	−9.9	451.8

*Sum of squares were calculated using Type II ANOVAs, and are displayed for each factor in the analysis, ground State and feature, as well as their interaction, model residuals and total model variation. Sum of squares are decomposed into the contributions by species turnover, intraspecific variability and their covariation. Covariation is calculated by subtracting both species turnover and intraspecific variability contributions from the Total column, and can be negative or positive. Values are bolded if significant (*P* < 0.05).*

**FIGURE 4:**
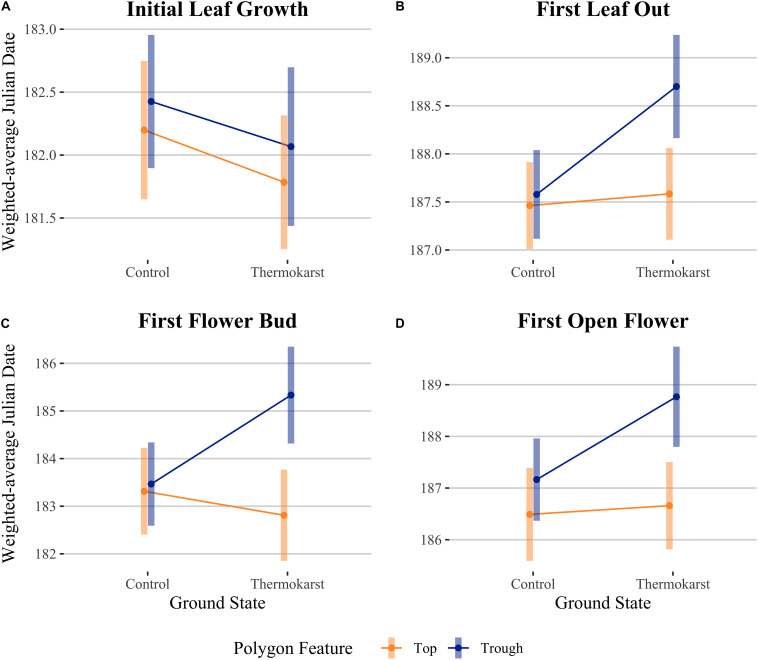
Interaction plots for estimated marginal means comparing ground state (control vs thermokarst) and polygon feature (top = orange vs trough = blue) categories, while controlling for cumulative thaw degree days (TDD). Comparisons are **(A)** initial leaf growth (TDD = 8.0), **(B)** first leaf out (TDD = 22.6), **(C)** first flower bud (TDD = 12.0) and **(D)** first open flower (TDD = 23.1). Points denote mean values, while bars represent 95% confidence intervals.

We evaluated whether cumulative ground temperature up to a phenological event could explain differences in our leaf or flowering phenophase differences across our site. We found that cumulative TDD was positively correlated with first leaf bud (df = 72, *R*^2^ = 0.216, *P*< 0.001), full leaf out (df = 68, *R*^2^ = 0.193, *P* < 0.001), flower bud (df = 65, *R*^2^ = 0.505, *P* < 0.001), and first open flowering dates (df = 61, *R*^2^ = 0.607, *P* < 0.001; panel B of Supplementary Figure S1). We also found that the predictors ground state and polygon feature explained residual variation after TDD was accounted for in an ANCOVA (Figure 4; see Table 3 for associated statistics). We found that there remained no effect of ground state or polygon feature while including TDD as a covariate for first leaf bud. The effect of site, type and their interaction were significant for first leaf out, with thermokarst troughs delayed compared to control tops (1.24 days (SE 0.35), *t*_65_ = −3.50, *P* = 0.005), thermokarst tops (1.12 days (SE 0.36), *t_65_* = −3.10, *P* = 0.017) and control troughs (1.12 days (SE 0.34), *t*_65_ = −3.27, *P* = 0.011). For first flower bud there was a direct effect of polygon feature and the interaction of feature and ground state, with thermokarst troughs also delayed as compared to control tops (2.02 days (SE 0.68), *t*_62_ = −3.00, *P* = 0.025), thermokarst tops (2.52 days (SE 0.70), *t*_62_ = −3.61, *P* = 0.004) and control troughs (1.87 days (SE 0.67), *t*_62_ = −2.78, *P* = 0.043). For first open flowers, we found that there was no significant interaction (as found when including only the categorical predictors), but that there was a direct effect of polygon feature and ground state. For this phenophase thermokarst troughs were delayed as compared to control tops [2.28 days (SE 0.66), *t*_58_ = −3.46, *P* = 0.006] and thermokarst tops [2.11 days (SE 0.64), *t*_58_ = −3.28, *P* = 0.011], but not significantly so for control troughs.

**TABLE 3:** Abundance-weighted Type II ANCOVA results for all four phenological timing response variables, including cumulative Thaw Degree Days (TDD) as a covariate.

**Response**	**Effect**	**SS**	***F***	***P***
Initial leaf growth	**TDD**	**27.5**	**19.553**	**<0.001**
	Ground state	2.7	1.912	0.171
	Polygon feature	1.1	0.779	0.381
	Interaction	0	0.001	0.922
First leaf out	**TDD**	**22.7**	**23.424**	**<0.001**
	**Ground state**	**6.2**	**6.392**	**0.013**
	**Polygon feature**	**5.1**	**5.229**	**0.025**
	**Interaction**	**4.3**	**4.415**	**0.040**
First flower bud	**TDD**	**264.6**	**73.167**	**<0.001**
	Ground state	7.4	2.049	0.157
	**Polygon feature**	**24.9**	**6.888**	**0.011**
	**Interaction**	**22.4**	**6.199**	**0.015**
First open flower	**TDD**	**205.8**	**73.125**	**<0.001**
	**Ground state**	**11.9**	**4.228**	**0.044**
	**Polygon feature**	**27.3**	**9.708**	**0.003**
	Interaction	6.9	2.454	0.122

*Significant *P-*values (<0.05) are in bold.*

**FIGURE 5:**
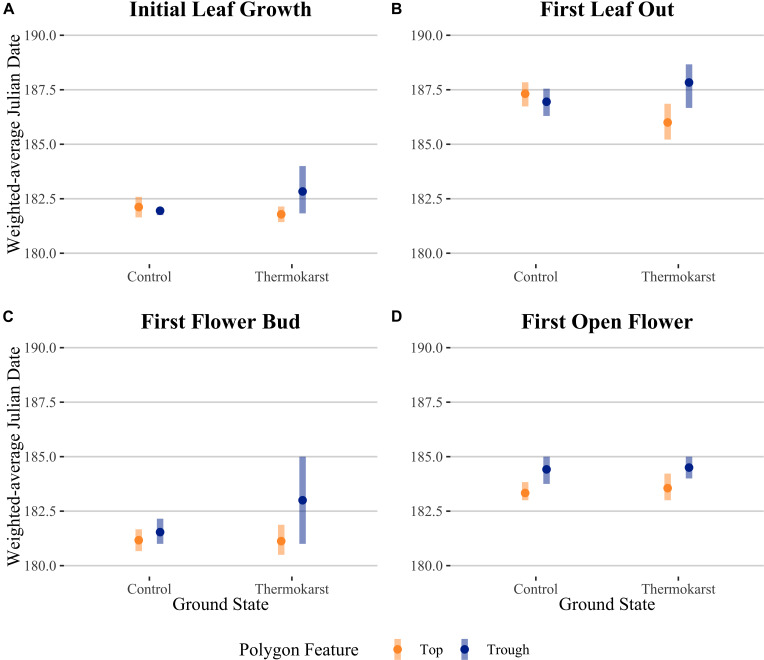
Julian dates for *Salix arctica* phenophases **(A)** initial leaf growth, **(B)** first leaf out, **(C)** first flower bud and **(D)** first open flower across ground state (control vs thermokarst), with colors denoting polygon feature (top = orange vs trough = blue) categories. Points denote mean values, while bars represent 95% confidence intervals.

In order to evaluate intraspecific variation in phenological responses across microtopography, we focused on the species *S. arctica* which was found to be widespread and abundant across both polygon feature and ground state (Figure 5). Both initial leaf growth and full leaf out phenophases showed significant interaction effects between ground state and feature (*F*_1,5__3_ = 6.0, *P* = 0.018, and *F*_1,5__5_ = 7.1, *P* = 0.010, respectively). However, after a pairwise comparison correction, initial leaf growth was only found to be marginally significantly delayed [1.05 days (SE 0.41)] compared to thermokarst tops (*t*_53_ = −2.54, *P* = 0.065). Leaf out was delayed 1.83 days (SE 0.69) in thermokarst troughs compared to thermokarst tops (*t*_55_ = 2.66, *P* = 0.049) but presented 1.32 days (SE 0.50) earlier in thermokarst tops than control tops, though this was also found to be only marginally significant (*t*_55_ = 2.64, *P* = 0.051). We found no effect of ground state or polygon feature on first flower bud date. Polygon feature was the predominant control on first open flowering in this species, with open flowers delayed 1.01 days (SE 0.45) in troughs compared to tops (*t*_25_ = −2.28, *P* = 0.031; *F*_1,25_ = 6.7, *P* = 0.016).

## Discussion

The delays we found in three key phenological traits for plant communities in thermokarst troughs demonstrate the impact of a geomorphological process that has largely been overlooked in ecology literature. Our results suggest that thermokarst, and the subsequent ground subsidence due to ice wedge degradation, decreases soil temperature and these altered abiotic conditions affect key phenophase timings. The differences we observed in plot-averaged phenophase traits are largely driven by turnover in community membership, and not intraspecific variability, which operate differently depending on microtopography. We found that ground state (thermokarst or non-thermokarst) and polygon feature (top or trough) explained residual variation in our models after accounting for temperature. This suggests that geomorphic features influence other environmental variables (such as soil moisture, active layer depth, snowpack accumulation, and spring runoff water collection; Jorgenson et al., 2015) important for predicting phenological events. Thermokarst trough communities experienced a 7–15% delay in phenological timing, which is contrary to most phenological warming predictions for the Arctic. While thermokarst requires the presence of ground ice to occur, the fact that it is present in up to 50% of the near-surface ground volume of the high Arctic points to a large area of the landscape that could experience these microtopographical changes (Pollard and French, 1980). Thus, while warming in stable areas is likely to advance plant phenology, concurrent and patchy changes to the landscape via subsidence may have delaying effects on phenology and community turnover.

Arctic phenology is predominantly driven by cues which vary inter-annually (e.g., temperature, snowmelt and soil thaw), vs cues which are invariant (e.g., photoperiod; Wheeler et al., 2015), and plant phenological timing tends to be more sensitive to these variations at colder sites (Prevéy et al., 2017). As these variant drivers are impacted by thermokarst, phenology in the Arctic may be particularly sensitive to ground processes. Interestingly, the delays we found in thermokarst trough phenology (1–3 days) are similar in magnitude, but opposite in direction to the 2–5 day advancement in phenology found over two decades in an OTC warming experiment at a nearby site on Ellesmere Island, Nunavut, Canada (Bjorkman et al., 2015). We caution that the results found in our study are from a single site over a single growing season, and while there are inherent limitations with these constraints, we suggest that this study can still provide insights into the often-overlooked effect of geomorphology on vegetation in the arctic. If thermokarst-induced changes to plant communities tend to delay phenology while drier sites experience earlier phenology [or no significant change, as found in control plots in Bjorkman et al. (2015)], we may see a broadening of overall Arctic phenological windows, based on the heterogeneous response of landscape microtopography due to climate warming. Thermokarst may delay flowering such that bud break and flower development occur later in the summer growing period for some species, while earlier flowering cued by early snow melt in non-thermokarst areas may increase risk of frost damage for flowers (Wheeler et al., 2015). Thermokarst-induced conditions tend to delay snowmelt timing, an important phenological driver which we were unable to assess in this study. This may contribute to an inequality of fitness in plant communities across microtopographies, further driving differences in community-level diversity and abundance across the landscape.

Given there are such large shifts in community membership between the microtopographies at our site, we used a relative site-based measure to compare these disparate communities. Recent work suggests that this approach is a powerful way to compare phenology across studies with dissimilar plant communities (Prevéy et al., 2017), such as those created by the effects of thermokarst. Site-level measures of phenology are an important way of quantifying how resource availabilities for pollinators and herbivores in the high Arctic may shift with climate change as both resident pollinators (Elberling and Olesen, 1999; Olesen and Jordano, 2002) and herbivores (Manseau and Gauthier, 2013) tend to be generalists capable of utilizing a suite of available plant life. However, as responses to climate change are often species- and functional group specific (Iler et al., 2013; Post et al., 2016) we need to better understand how intraspecific variability and species turnover both contribute to average phenological change in plant communities. Our results suggest that phenology is largely delayed at the plot-level for thermokarst troughs because the changed abiotic conditions act as environmental filters which favor shifts in plant community membership. In certain cases, such as for the first leaf bud and first open flower phenophases, we see a negative covariance in the direct effect of ground state or feature. Here, turnover and intraspecific variability are selecting for opposing (early vs late) phenological dates, which may explain why there are few significant direct effects of polygon feature or ground state. Examining a dominant species at our site, our analysis of intraspecific variation demonstrates that *S. arctica* does shift phenophase timings in response to the changed abiotic conditions associated with ice wedge thermokarst (i.e., in general, thermokarst delays phenophase timings). However, this effect is not consistent across phenophase traits and for first leaf out can depend on the polygon feature a community is found in (e.g., thermokarst tops found an advancement in timing compared to non-thermokarst terrain).

The melting of ground ice has a multitude of effects on the ecology and vegetation of the high Arctic (Billings and Peterson, 1980). However, to our knowledge, climate-induced changes to geomorphic features have yet to be considered when projecting phenological change in a warming Arctic. The changed hydrological pattern of thermokarst results in colder and more stable ground temperatures due to increased vegetation cover, which has a dampening effect on both daily and seasonal temperature variation (Shur and Jorgenson, 2007; Becker and Pollard, 2016). The species found colonizing areas undergoing thermokarst are likely drawn from the local high Arctic species pool already present in neighboring wetland areas (Cooper et al., 2004), and these locally adapted wetland species could be timed to complete life history processes within the narrow summer window despite colder summer conditions imposed by thermokarst processes (Becker and Pollard, 2016). The cooling effects of thermokarst-altered abiotic conditions might offset effects of warming air temperatures, similar to that of later snow melt (Wipf and Rixen, 2010), and are difficult or impossible to replicate using experimental warming studies. It is clear that there are multiple drivers of ecological change in the high Arctic, and an acknowledgment of the interaction between warming temperatures and geomorphological processes may alter our predictions of how climate change will impact this region.

Perhaps the greatest concern of climate change effects on high Arctic phenology is trophic mismatch, which is a lack of synchrony between the life cycle timing of consumers and their resources (Durant et al., 2007). In the case of the high Arctic, many organisms are largely transient either as resident insect pollinators with short summer life cycles, or migratory birds (McKinnon et al., 2012). Deepening snow conditions delay insect emergence (Høye and Forchhammer, 2008), and as ice wedge thermokarst leads to trough subsidence, and thereby a deeper snowpack, this suggests delayed insect emergence for colonies within troughs. Interestingly, a delayed vegetation phenology in troughs but advancement in non-thermokarst areas may lead to an overall broadening of the window for plant provisions that insects rely on. Insects that emerge late will not benefit from this, but insects that advance their timings in concordance with non-thermokarst terrain may see a net benefit in a longer period of resource availability. Many migratory birds nest in the Arctic and their reproductive timings are tuned to account for peak plant nutrition. For example, some species such as Snow Geese, *Chen caerulescens*, are only able to partially adjust their breeding phenology to compensate for availability of high-quality food and timing mismatches result in poor gosling growth (Doiron et al., 2015). Our leaf phenophase results suggest a delayed timing for peak green-up in thermokarst trough communities, which are one of the more productive wetland habitats available to these organisms in the polar desert climate. This also infers a later date for peak leaf-nitrogen, an important food source for geese (Chapin, 1980). Additionally, our results show thermokarst-induced trough community reshuffling resulted in higher biomass of *Dupontia* and *Eriophorum* species – resources preferred by growing chicks (Manseau and Gauthier, 2013). Given the prevalence of thermally sensitive ground ice in the high Arctic, the cumulative effects of shifting phenological timing on trophic mismatch should be investigated further.

## Conclusion

The geomorphology and ecology of the high Arctic are tightly interconnected and a warming climate is predicted to affect both greatly. Given the prevalence of thermally sensitive ground ice and the resulting abiotic changes that occur due to thermokarst, it is essential that we consider this geomorphological process when predicting the effects of climate change on plant phenology. Due to the patchy but significant distribution of ground ice, at a landscape level we are likely to see two divergent phenological results as a consequence of climate change: (1) delayed phenology of communities overlaying thermokarst-affected ice wedge troughs and (2) an increased divergence in phenological timings between polygon features. We emphasize the necessity of considering geomorphological change in studies of the effects of climate change on plant communities found in areas underlain by permafrost, as these ground states underlie a large portion of the high Arctic landscape and are particularly susceptible to the effects of climate warming. Additionally, we suggest that the bridging of historically disparate natural sciences will improve on our ability to predict the effects of climate change, or at the very least, increase our understanding of the underlying intricacies of natural systems.

## Data Availability Statement

Soil temperature and phenological observation data used in this publication are available from the Figshare repository (10.6084/m9.figshare.12248312).

## Author Contributions

CC and MB conceived of and conducted the field work for this project. CC analyzed the data. CC and MB wrote the manuscript. All authors contributed extensively to the drafts and approved the final manuscript.

## Conflict of Interest

The authors declare that the research was conducted in the absence of any commercial or financial relationships that could be construed as a potential conflict of interest.
