# Supplementary material for: The Importance of Incorporating Landscape Change for Predictions of Climate-Induced Plant Phenological Shifts
Source: Front Plant Sci. 2020 Jun 25;11:759. doi: 10.3389/fpls.2020.00759 (PMC7329987; doi:10.3389/fpls.2020.00759)
Supplement: Supplementary file 1 [file Data_Sheet_1.pdf]

Supplemental Materials

Table S1. Species list of all vascular plants used in this analysis, with associated family names and shortened species codes. Ascension number refers to voucher collection submitted to the Marie-Victorin Herbarium in Montreal, Quebec, Canada.

Species Name	Family	Species Code	Ascension Number
Alopecurus magellanicus	Poaceae	alo.mag	187791
Arctagrostis latifolia subsp. *latifolia*	Poaceae	arc.lat	187815
Carex aquatilis var. *minor*	Cyperaceae	car.aqu	187792
Carex rupestris	Cyperaceae	car.rup	187808
Cerastium arcticum	Caryophyllaceae	cer.arc	187793
Draba arctica	Brassicaceae	dra.arc	187795
Dryas integrifolia	Rosaceae	dry.int	187801
Dupontia fisheri	Poaceae	dup.fis	187817
Elymus alaskanus	Poaceae	ely.ala	187819
Eriophorum triste	Cyperaceae	eri.tri	187803
Eriophorum scheuchzeri	Cyperaceae	eri.sch	187804
Festuca brachyphylla var. *brachyphylla*	Poaceae	fes.bra	187818
Luzula confusa	Juncaceae	luz.con	187809
Papaver radicatum var. *radicatum*	Papaveraceae	pap.rad	187798
Pedicularis hirsuta	Orobanchaceae	ped.hir	187812
Poa pratensis subsp. *alpigena*	Poaceae	poa.pra	187820
Potentilla arenosa	Rosaceae	pot.are	187797
Salix arctica	Salicaceae	sal.arc	187802
Saxifraga cernua	Saxifragaceae	sax.cer	187816
Saxifraga tricuspidata	Saxifragaceae	sax.tri	187821
Silene involucrata subsp. *involucrata*	Caryophyllaceae	sil.inv	187794
Stellaria longipes subsp. *longipes*	Caryophyllaceae	ste.lon	187811

Table S2. Abundance-weighted linear mixed effects model results for all four phenological timing response variables. Significant *P* values (< 0.05) are in bold.

Response	Effect	DF	*F*	*P*
**Initial leaf growth**	Ground State	67	3.4	0.071
	Feature	67	0	0.892
	Interaction	67	0.1	0.730
**Full leaves**	**Ground State**	**63**	**4.6**	**0.036**
	Feature	63	0.5	0.501
	**Interaction**	**63**	**4.5**	**0.039**
**Flower bud**	Ground State	60	0.2	0.679
	Feature	60	2.4	0.123
	**Interaction**	**60**	**8.1**	**0.006**
**Open flowers**	Ground State	56	0.5	0.487
	Feature	56	0.3	0.557
	**Interaction**	**56**	**9.3**	**0.003**

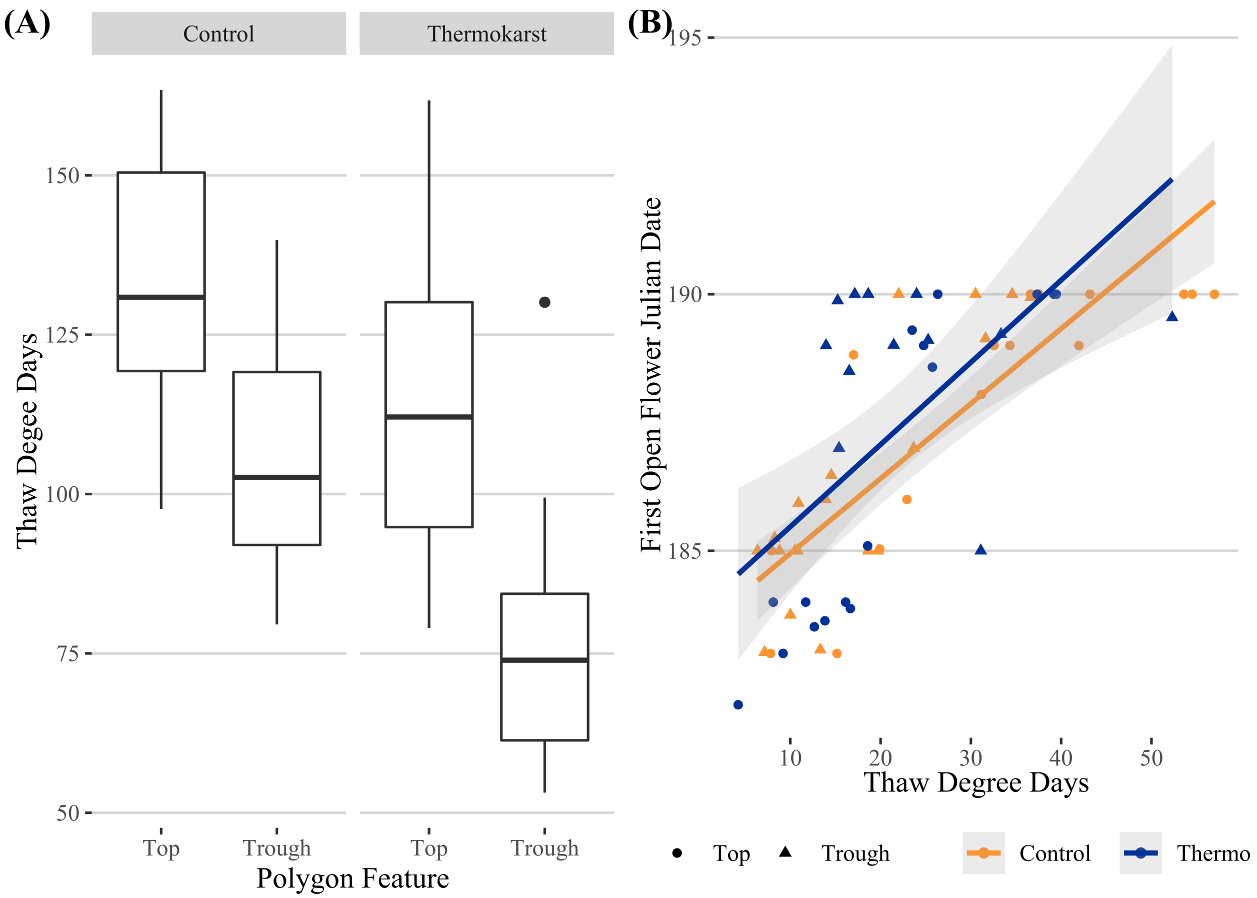

Figure S1. (A) Thaw Degree Days (TDD) at 10 cm soil depth at each plot for the study season. Thermokarst plots experienced significantly lower ground temperatures for the study period than control areas ($F_{1,70} = 23.777$, $P < 0.001$), with polygon troughs having lower temperatures than polygon tops ($F_{1,70} = 41.173$, $P < 0.001$). No significant interaction effect was present. Box whiskers represent 1.5 interquartile range (IQR) boundaries. (B) First flowering Julian date (open flowers) as predicted by cumulative thaw degree days. Shapes depict polygon features (tops vs. troughs) and colored lines depicting the linear regression slope of the model fitted separately for ground states (control vs. thermokarst). Shaded regions depict the standard error for each model. First flowering dates occurred inclusively between Julian dates 179 and 190 across all plots.

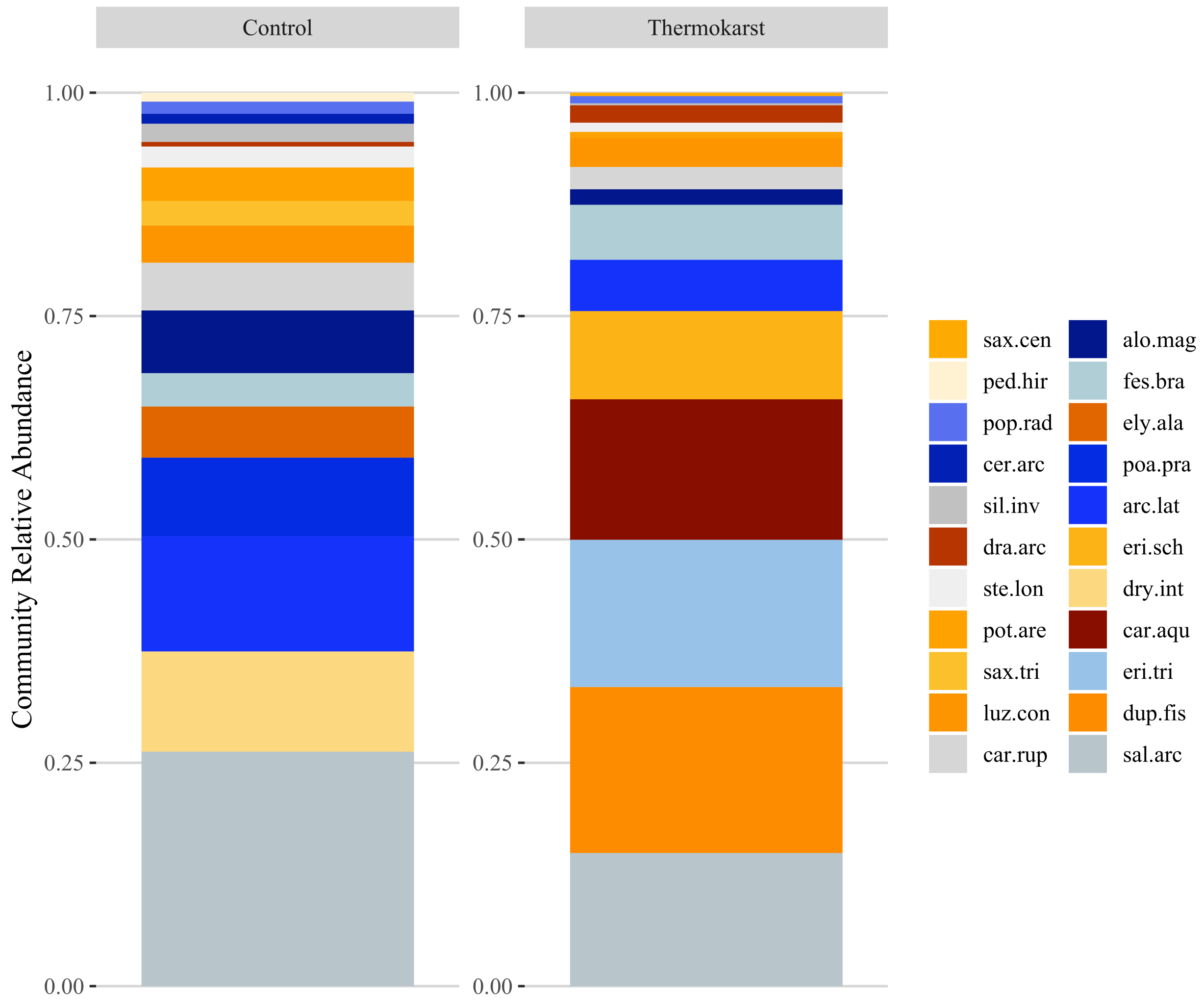

Figure S2. Relative abundance of species found across ground state (polar desert/control and thermokarst), determined by percent cover estimates. Species names are displayed as species codes, with associated Latin names found in Table S1.

Methods S1. Variance Decomposition

For each phenophase trait, we calculated average Julian dates weighted by plant species relative abundances. In order to disentangle the contribution of both intraspecific variability and species turnover to the differences found in phenophase dates across our study site, we calculated two averages per plot. Equation 1 calculates a *fixed average* whereby each plot value is a weighted average (p_i, or relative abundance) of the species mean Julian date across the entire study site ($x_{i\_species}$). This equation ignores intraspecific variability in phenology, and as such the Julian date for each species is the same (mean) value in every plot, and thus the differences between plots are driven by species composition and relative abundance solely.

Eq. 1:

$$Fixed\ average = \sum_{i=1}^{s} p_i x_{i\_species}$$

Equation 2 calculates a *specific average* whereby each plot value is a weighted average (p_i, or relative abundance) of the plot-specific Julian dates observed for that species (x_{i_plot}). This average incorporates the intraspecific variability of phenophase dates within species, in which differences between plots are driven by both species composition/abundance and intraspecific variability caused by habitat differences.

Eq. 2:

$$Specific\ average = \sum_{i=1}^{S} p_i x_{i\_plot}$$

We then decomposed variation in phenophase traits following Lepš et al. (2011). The difference between the fixed average and specific average gives us the contribution of intraspecific variability (Equation 3).

Eq. 3

$$Intraspecific\ variability = Specific\ average - Fixed\ average$$

We use these three community trait parameters (specific and fixed averages, and the difference between them) as response variables in separate ANOVA analyses explained by the factors ground state (polar desert vs. control) and polygon feature (top vs. trough). We calculate the total Sum of Squares (SS) in each of the individual analyses and use this as the total variability explained by each component (SS_{fixed}, $SS_{specific}$, SS_{intra}) of community-weighted trait means. The SS for each ANOVA is further decomposed into the variability explained by each of our explanatory variables, as well as any unexplained variation (error).

We use the total variation explained in the specific averages as our total variation for the community trait means ($SS_{specific}$).

If the effects due to species turnover (SS_{fixed}) and intraspecific variability (SS_{intra}) vary independently, their values sum to $SS_{specific}$. If these two effects are negatively correlated, $SS_{specific}$ will be lower than if the two effects are independent (or higher if they are positively correlated). This refers to the effect of covariation, which is described in Equation 4. This can occur if, for example, species develop earlier phenology in plots dominated by species with early phenology, and later phenology in plots dominated by species with late phenology in general (positive covariation).

Eq. 4:

$$SS_{cov} = SS_{specific} - SS_{fixed} - SS_{intra}$$

In summary, we decompose the effects of species turnover and intraspecific variability and their covariation on the total variation explained, as well as for each individual model term, as found in Table 2 of the manuscript.
